# Understanding the Paths to Appearance- and Performance-Enhancing Drug Use in Bodybuilding

**DOI:** 10.3389/fpsyg.2018.01431

**Published:** 2018-08-08

**Authors:** Ronan Coquet, Peggy Roussel, Fabien Ohl

**Affiliations:** ^1^Institute of Sport Sciences of the University of Lausanne, Faculty of Social and Political Sciences, University of Lausanne, Lausanne, Switzerland; ^2^Aix-Marseille University, CNRS, ISM, Marseille, France; ^3^Laboratory Capitalism, Culture & Society, Institute of Sport Sciences of the University of Lausanne, Faculty of Social and Political Science, University of Lausanne, Lausanne, Switzerland

**Keywords:** doping in sports, career, moral disengagement, bodybuilding, appearance and performance enhancing drug, interactions, qualitative methods

## Abstract

How do gym-goers who are normally not inclined to resort to appearance- and performance-enhancing drugs (APEDs) progressively normalize their use? Based on data collected through a year and a half of participant observation in a gym and 30 semi-directive interviews with practitioners with varying profiles in French-speaking Switzerland, this article examines the evolution of practitioners’ relations with APED use by articulating various levels of analysis. Associated with social vulnerabilities, the progressive normalization of APED use is concomitant with the “conversion” to bodybuilding. Our results show the extent to which and under what conditions interactions within the layout of gyms can influence practices. From refusal to normalization, our results suggest that APEDs and the associated beliefs coincide with career stages, which we aim to bring to light here.

## Introduction

Various disciplines, such as history, sociology, and psychology, have addressed the question of doping, each with its own methods and theoretical frameworks. However, the knowledge that each discipline produces is generally discussed within only that disciplinary field. The understanding of a complex topic such as appearance- and performance-enhancing drug (APED) use would, however, benefit from exchanges across disciplines since the conditions that make doping behaviors possible are simultaneously historical, economic, social, and psychological. It therefore seems useful to try to articulate the conditions to better understand APED use. The aim of this article is to pursue this approach by analyzing the case of bodybuilding. The perspective that it adopts makes it possible to focus attention on the effects of social situations, life courses, interactions, socializations, and experiences that may explain the processes leading to APED use. Our more “ecological" approach and our qualitative methods are complementary to quantitative psychological analyses centered on individual variables (personality disorders, appearance pathologies, and motivational variables). Sociology studies on bodybuilding have described bodybuilders’ lifestyles (diets, training, and financial sacrifices), quests and beliefs, differentiated uses of doping substances and justifications for their use (Klein, 1993). We suggest that the articulation of different disciplinary approaches could be relevant, especially a discussion of the mechanisms of moral disengagement, including the matter of the sliding scale identified by Boardley and Grix (). While moral disengagement is most often analyzed with respect to specific times and situations, we argue that it is mainly a process constructed step by step on the basis of a series of interactions. One hypothesis is that interactions can produce a “conversion" among practitioners. This is expressed in individuals’ perceptions and interpretations of the meanings of practices, which result in an adherence to the bodybuilding subculture. In other words, as with religious beliefs, converts adhere to a belief in the value of the practice, a kind of doxa, that corresponds to the core values and beliefs of a field, a type of orthodoxy that is “accepted as self-evident" (Bourdieu, 1984b, p. 471), and these core values and beliefs fix the norms of desirable practices. It explains how this “obsession" with muscle is constructed over time with, as its corollary, a normalization of APED use despite the objective risks. A second hypothesis is that the outcomes of interactions depend on gym-goers’ social dispositions and social vulnerability that characterize them when entering a gym. This combination may transform the initial motives of practice and can drive morality changes. Therefore, it is crucial to understand what differentiates the trajectories of ordinary gym-goers who are involved in the gym and training from bodybuilders who dedicate their entire lives to bodybuilding (in terms of amount of training, presence at the gym, nutrition, competition, social networks, lifestyle, etc.). None of the bodybuilders we met in our study had intended *a priori* to use doping substances. They are ordinary people who became bodybuilders and APED users. How one becomes a bodybuilder and decides to use APEDs is interesting to analyze because the quest for muscle volume is often not understood.

## Apeds and Bodybuilding

### The Need for an Approach Beyond Pathologizing and Moralizing Analyses of Bodybuilding

The literature on bodybuilding provides two focal orientations in the field of psychology. The first analyzes bodybuilders’ drug consumption through the lens of a pathological desire to become muscular. Bodybuilders display obsessive-compulsive disorder (Pope et al., 2000), behavioral addiction (Berczik et al., 2012), mental health problems (Wolke and Sapouna, 2008), and a body dysmorphic disorder/body image disorder labeled muscle dysmorphia or bigorexia (Pope et al., 1993).

A second set of studies focuses on psychosocial predictors of doping intentions and moral disengagement. Researchers have observed the prevalence of doping in sports, and many of them have focused on risk factors for anabolic steroid use amongst bodybuilders (Blouin and Goldfield, 1995; Hildebrandt et al., 2007; Ntoumanis et al., 2014). These authors show that doping intentions and doping use are significantly related to dissatisfaction with body appearance. Not surprisingly, there is a high prevalence of doping (77.8%) among competitive bodybuilders (Blouin and Goldfield, 1995). Some of the psychological research studies bodybuilding without focusing on the “obsession with muscle.” They explain the drive for muscularity (Schneider et al., 2016) or APED uses (Petroczi and Naughton, 2008; Bahrami et al., 2014) through psychological variables (Hurst et al., 2000; Emini and Bond, 2014) and integrative socio-cognitive approaches (Wiefferink et al., 2007). They use models that do not present a pathological view of bodybuilding. However, prevalence measures and the identification of gateways are based on descriptions and correlations with regard to mental health concerns and are often used to justify research on doping. Prevalence studies can be ambivalent: they tackle public health issues and challenge researchers (Sagoe et al., 2014). Some authors show that in the background of epidemiological research, there is a risk of a moralistic approach to analyzing bodybuilders’ behavior (e.g., Kartakoullis et al., 2008; Christiansen et al., 2017), and the same risk exists with qualitative studies that analyze consumers’ discourses to understand how they normalize doping and risks (Monaghan, 2001; Stewart and Smith, 2008; Boardley and Grix, 2014). As a consequence, and bearing in mind the historical dimension of the definitions of the normal and the pathological (Canguilhem, 2012), we assume that considering extreme muscle solely as a pathology is too narrow, and we suggest the exclusion of moral judgments when analyzing the bodybuilding subculture.

### Analyzing the Meaning of APEDs for Bodybuilders

Despite this interesting set of studies, it is still difficult to explain how ordinary people become bodybuilders, as we have observed the phenomenon. Additionally, research on moral disengagement mainly focuses on how judgments may explain why bodybuilders use APEDs. Taking care of the temporality of bodybuilders’ careers allows us to simultaneously discuss the pathological diagnosis established and identify the roots of what could be viewed as moral disengagement. Aiming to complement these previous studies, we argue that it is necessary to provide more qualitative data for an in-depth understanding of how judgments are shaped over time. We agree that sense-making related to body image better explains APED uses than a psychopathology-oriented approach (Hauw and Bilard, 2017), and we suggest that bodybuilders’ APED use is the outcome of a combination of individual properties (body image, social background, and trajectory) and social interactions in the context of the gym. Attention to these processes, the relations between bodybuilders and the contexts is essential for understanding why it make sense for bodybuilders to use enhancing substances.

#### Widespread Body Dissatisfaction but Few Bodybuilders

**FIGURE 1:**
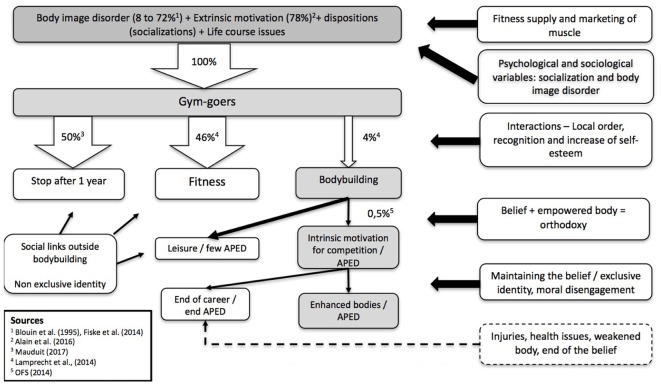
The process of becoming APED user in bodybuilding.

People may not express their pathologies in their lifestyles, and if they do, they could use many other means to express or overcome their low self-esteem and body dissatisfaction, including using anabolic–androgenic steroids (AAS) without becoming bodybuilders (Griffiths et al., 2017). This is why, compared to the usual high rate of body dissatisfaction (8–72%), bodybuilders (0.5%) represent only a tiny share of the 16% of the Swiss population who belong to a gym (see **Figure 1**). Joining a gym is often motivated by the wish to reshape the body to be in conformity with aesthetic, hygienic or sports norms (Hurst et al., 2000; Baghurst and Lirgg, 2009; Goldfield, 2009; Boardley and Grix, 2014). Even APED use is based on a diversity of motivations (Christiansen et al., 2017). AAS users may (1) have a “fascination with the effects of pharmacological substances on human physiology,” (2) be motivated by a desire for well-being and to look and feel good, (3) want to enjoy life fully “even if that entails taking risks,” and (4) want to “prepare for and perform at competitions” (in the case of athletes).

However, most of the studies focus on the outcomes, or how bodybuilders behave, but few focus on the processes that explain the behaviors. Our observations do not reveal specific psychopathologies among bodybuilders. Their investment both in the gym and outside the gym is rather the expression of their lifestyles, expertise, performance, etc. Thus, bodybuilders are not really different from other top-level athletes. They learn to control everything in their lives and give attention to nutrition, training, sex, sleeping, recovering, drugs, etc. Though it seems sometimes obsessional, similar to a perfectionist attitude, it is this same perfectionism that incites bodybuilders to take risks to be successful (Schnell et al., 2014). Although low self-esteem and body dissatisfaction seem to be a common background (Hildebrandt et al., 2006) that is shared with a significant part of the population, it is also important to understand the crucial role that the investment in bodybuilding plays in individuals’ behaviors and what bodybuilding means for them (Hauw and Bilard, 2017). Indeed, at least in our sample, none of the interviewees wanted to become bodybuilders when they first entered the gym, and none of them believed later that their nutrition, training and doping was without any risk and would give them completely healthy bodies and eternal youth. Therefore, the choice of a bodybuilder lifestyle, which places such importance on nutrition and training, monopolizing time and space, may not be explained by pre-existing psychological characteristics alone.

#### The Performative Framework of the Gym

In the current broad fitness industry (Sassatelli, 2010), bodybuilding represents a subculture with its own history. The purpose here is not to present an exhaustive history of bodybuilding but, rather, its main consequence: the creation of a specific social context that incites some gym-goers to become bodybuilders and experience body enhancement through APED. Thus, we suggest a focus on how the gym frames gym-goers’ experiences. Frame analyzing in the sense of (Goffman, 1974) is “a step toward unpacking the idea of context” (Scheff, 2005, p. 368) because our social experiences and perceptions of the environment are dependent on social context. The gym is a social space whose meaning is under the influence of a combination of images, discourses and figures associated with it. Therefore, it is important to recall some key historic, economic and social characteristics of bodybuilding to explain how they influence individuals’ experiences and facilitate bodybuilders’ moral and behavioral changes. Bodybuilding spread as a (sub)culture and a market over the course of the 20th century because of economic operators that shrewdly promoted it. Key factors in the success of this muscle culture include efficient marketing, the creation of organizations in charge of competition and a high density of local actors and facilities to make the supply easily accessible. Among Weider’s family legacy is the value given to muscles. Through a coherent sales strategy, they commercialized the muscle and made it desirable. They sold the promise of a perfect body and a belief in the value of bodybuilding by creating magazines (e.g., *Your Physique* in 1940) and offering gyms, courses, nutrition advice, etc., that contributed to the spread of the “ideology” (Klein, 1993, p. 87). Since Weider’s first steps, a large number of media, movies and competitions have increased the legitimacy of muscle and, as a corollary, the use of APEDs in bodybuilding. Bodybuilding’s success was also supported by an idealization of muscles as a strong symbol of masculinity (Monaghan and Atkinson, 2014). The Weider family also played a key role in the organization of bodybuilding. They founded the International Federation of Bodybuilding (IFBB) in 1946 and created Mr. Olympia, a key event in the discipline. Along with the Weiders’ initiatives, numerous epigones developed the bodybuilding market, including drugs for which “gym owners or managers and bodybuilding instructors work most often as retailers” (Paoli and Donati, 2014, p. 66). They are particularly efficient because they are frequently user-dealers (Paoli and Donati, 2014), which helps them to be recognized as experts. Trading the same doping products that they consume also reinforces them as opinion leaders of the bodybuilding lifestyle.

The importance of the success of the fitness industry as a mass leisure activity with new practices, more suppliers, and a growing and important economy, changed the cultural meaning of muscle and the norms of what the male body can be. The large supply of products, successful marketing and multi-channel communication supported the dissemination of bodybuilding in many countries. Although some bodybuilders, such as the high-level competitors in Europe, are still perceived as a “kind of freak” (Monaghan, 2001, p. 25), the bodybuilding subculture seems to be less extraordinary today than before. Treating bodybuilding as a pathology of appearances is definitely too narrow of an approach to understanding why people become involved in it.

#### Understanding Bodybuilders Instead of Demonizing Them

Despite its accessibility, the bodybuilding subculture is still perceived as deviant, at least because of the antidoping norms (Petróczi, 2007). However, in many cases, APED use is not a real concern in bodybuilding; the prevalence of APED use among competitive bodybuilders is high, and doping is not perceived as a real moral issue. APEDs are no longer confined to competitive bodybuilders (Griffiths et al., 2017) as they were in the 1960s (Lazuras et al., 2017). Even the meanings associated with APEDs have changed over time. AAS are sometimes presented in some countries as an ordinary enhancing anti-aging resource.

This is why the perception of bodybuilders is ambivalent: their consumption is condemned by the antidoping code but also valorized outside the sport. Antidoping organizations make the bodybuilder a deviant, independent of the potential pathologies. Researchers also influence the external identity of bodybuilders and sometimes spread an image of practitioners unaware of the risks. By stressing high prevalence rates, or dramatically exaggerating the consumption of doping products, researchers may contribute to a moral panic (Critcher, 2014). The substantial scientific literature that associates bodybuilding and pathologies may justify the research and its funding, but it also demonizes the bodybuilding drug subculture. Unsurprisingly, the literature on bodybuilding has been criticized for the stereotypical view of the bodybuilding subculture that it offers (Monaghan, 2001).

It is important to bear in mind for a critical analysis of research that instead of demonizing bodybuilders, one should try to explain why they give so much importance to their body appearance and engage in a bodybuilding career. There is a gap between bodybuilding as a subcultural practice, with its specific social context, and the literature on the individual, which suggests that pathologies such as dysmorphic disorder/body image disorder are the key explanations for why someone becomes a bodybuilder. For this reason, to understand why gym-goers invest themselves in a bodybuilding career, it is important to view them as ordinary people and to focus on what occurs within the gym. Experiences including going to the gym, interacting with other gym-goers, having relationships with trainers and staff, and being recognized and valorized support one’s entry into a bodybuilding career. It is crucial to study the processes that change the practices in the gym and to give attention to the different stages of bodybuilders’ careers. Our analysis is centered on the notion of *conversion*, which enables a better understanding of what it is in bodybuilders’ careers that produces the adoption of beliefs and practices. Moreover, although some authors mention the gradual process of socialization into the bodybuilding subculture, none view that process as the key factor that explains how ordinary people become bodybuilders. It is a promising perspective that has already been explored with respect to anorexia by Darmon (2009) to understand the socialization process, and especially the role of interactions within the family and among peers. Therefore, our ambition is to understand why people who were not initially willing to have very large muscles or to consume drugs gradually normalize doping despite the evidence-based medical warnings of which they are all aware (e.g., Bonetti et al., 2008; Herlitz et al., 2010).

## Materials and Methods

Our study is based on data collected through participant observation and semi-directive interviews with gym-goers in French-speaking Switzerland.

### Participant Observation

Immersion in the gym lasted a year and a half and comprised approximately 100 field observations on different days of the week and at different times of day. Each session gave rise to a transcript of the observations. The duration of participant observation was determined by a principle of saturation, i.e., it was stopped when the information obtained became redundant. The choice of this method of investigation is justified for “understanding a particular organization or substantive problem” (Becker, 1958, p. 652). These sessions enabled us to collect qualitative data *in situ* on the practitioners’ interactions. Rather than pretending to be interested in this physical activity or striving to build a relationship of proximity with our interviewees *in situ*, we opted to maintain a role of observer-as-participant (Gold, 1958) so as not to disturb the course of the practitioners’ sessions.

The interviewer was not familiar with bodybuilding. He trained in a gym for the data collection but did not take drugs. His muscle volume was far below bodybuilder muscle standards. Therefore, he was an outsider and was perceived and trained as an outsider. He felt the distance and sometimes the disdain of the “real” bodybuilders and experienced barriers that he would need to overcome before being recognized. This is a very different position than that of other authors who used their heavily muscled appearances to be accepted “as part of the ‘serious’ bodybuilding community in the gym” (Boardley and Grix, 2014). Although trust and acceptance can be an advantage for discussion in the gym, being an outsider allows one to observe the symbolic barriers to achieving insider status and, before that, the steps required to be recognized as an acceptable peer. Having weak muscles and observing those who are earning the right to be recognized as they gain muscle brings advantages for observations of the processes through which gym-goers are recognized, included, and “elected” as insiders. Feeling belittled in the gym makes it possible to experience for oneself the symbolic boundaries that separate the different groups of practitioners (Andrews et al., 2005). However, with this outsider status, the study of substance use remains difficult for the researcher because, as a “deviant” practice, there is secrecy (Becker, 1963). This outsider status did not make it possible to have direct access to the backstage practices (Hughes, 1971). We therefore used the method of semi-directive interviews to try to identify the contexts and socialization frameworks that favor APED use in the study participants’ accounts.

### Semi-directive Interviews

We carried out 30 semi-directive interviews with gym-goers with varied characteristics in terms of age (21- to 59-years-old), gender (23 men, 7 women), occupation, marital status, and length of time in the practice (between 3 and 40 years, see **Table 1**). We differentiate ordinary gym-goers and bodybuilders. The latter are converted, which means that bodybuilding is their main occupation. It is a key element of their lifestyles, and most of them compete and use APEDs. Our informants were mainly recruited through third parties; others were contacted spontaneously, either by telephone, or face to face outside the time of participant observations, and then, with a snowball effect, these connections were extended into distinct networks. The interviews were conducted, as best suited the interviewees, in gyms, cafés or restaurants, parks or their homes, and each lasted between 1 and 3 h. To encourage an explanation of practices and of the unfolding of careers, we focused our interviews on “how” rather than “why” (Becker, 1998). The content of the interviews, which were recorded and then transcribed in their entirety, concerned the motives for engaging in physical activity in the gym and the ways in which one engages in dietary or pharmacological practices and the representations associated with these practices and with the evolution of sporting careers, life courses, and the social situations outside the gym that best account for the scale of the change in the practices and representations of some of our interviewees. These interview guidelines illustrate our intention to integrate APEDs as one dimension of our interviewees’ careers.

**Table 1:** Presentation of the interviewees.

Fictitious names	Age	Gender	Occupations	Years of experience in the gym	Average of training sessions per week	Investment in the gym	Gym enthusiasts status^∗^	Supplements ^∗∗^	APED ^∗∗^
Alex	21	Male	Safety officer	5	5.5	regular	convert – C	x	x
Alfred	31	Male	Unemployed	10	5	variable	convert – I	x	x
Bertrand	30	Male	Administrator	10	4	variable	ordinary	x	
Corentin	26	Male	Student	10	2.5	variable	ordinary	x	
Diego	30	Male	Nurse	4	3.5	regular	ordinary	x	
Eric	30	Male	Student	10	4	variable	ordinary	x	
Fabrice	35	Male	Policeman	20	5	regular	convert – C	x	
Guillaume	35	Male	Safety officer	6	5	regular	convert – C	x	x
Hervé	25	Male	Student	8	5	regular	ordinary	x	
Igor	39	Male	Administrator	20	4.5	regular	convert – I	x	x
Jean	33	Male	Teacher	17	5.5	regular	ordinary	x	
Kevin	25	Male	Executive director	6	5	variable	convert – I	x	
Léo	47	Male	Gym owner	17	4	regulier	convert – I	x	x
Marc	30	Male	Administrator	10	3	variable	ordinary	x	
Norbert	31	Male	Engineer	4	3	variable	ordinary	x	
Aude	49	Female	Administrator	30	3.5	regular	convert – C	x	x
Béatrice	40	Female	Administrator	20	5	regular	convert – I	x	x
Carole	30	Female	Commercial employee	3	4	regular	ordinary	x	
Dolorès	40	Female	Administrator	10	6	regular	convert – I	x	x
Pierrick	36	Male	Fitness instructor	20	5	regular	convert – I	x	x
Emilie	26	Female	Teacher	7	5	regular	ordinary	x	
Quentin	27	Male	Safety officer	11	4	regular	convert – C	x	
Romain	36	Male	Employee	20	5.5	regular	ordinary	x	
Samuel	59	Male	Gym owner	35	5	regular	convert – I	x	
Florence	37	Female	Fitness instructor	18	6	regular	convert – C	x	
Thierry	33	Male	Commercial employee	10	4	variable	ordinary	x	
Ubert	59	Male	Executive director	40	4	regular	convert – I	x	x
Gwenola	48	Female	Safety officer	19	6	regular	convert – I	x	x
Victor	26	Male	Employee	3	5	regular	Ordinary	x	

*^∗^For convert gym enthusiasts, we precise “C” for consonant conversion or “I” for introspective conversion. ^∗∗^Use or has used supplements or APED.*

### Content Analysis

With the aid of NVivo software, we carried out a systematic comparative content analysis of the interviews based on a coding of the various themes previously mentioned. Our work of *abductive analysis* (Timmermans and Tavory, 2012) was done in two main stages. In the first stage, a thematic analysis was used to characterize and dissociate the two different profiles. To this end, rather than analyze our interviewees’ retrospective *life stories* for what they are, we tried to understand how they were produced by reinserting them in moments of the participants’ careers so as to consider the effect of the *biographical illusion* (Bourdieu, 1986) and draw *causal inferences* (Katz, 2001). The correlation of the different levels of analysis – namely, perceived bodily sensations, interactions in the gym, and external social configurations in which our interviewees move – proved particularly favorable for identifying the adoption of pharmacological practices. The originality of this approach and the possibility provided by our sample population to compare, in the second stage of analysis, the careers of users and non-users of doping substances made it possible to identify the ways in which the trajectories of doping substance users might differ from those of ordinary gym users.

## Results

To explain the weakening of resistance to substance use, some psychologists refer to the mechanisms of moral disengagement (Boardley and Grix, 2014). Our results are consistent with work in this area but suggest a complementary view, showing that the combination of social factors, which play a decisive role in the taste for strength-building, and situational and contextual factors will “convert” individuals to bodybuilding and thus change their practices and their representations. However, although many studies have focused on the subculture of bodybuilding, few have addressed the essential question of how conversion takes place.

### Conversion to Bodybuilding

Not all practitioners become bodybuilders, and for those who do, it takes time. From gym-goers’ ordinary practice to the quasi-professional practice of bodybuilding, we differentiate a group of heterodox individuals (14) and a group of converts to bodybuilding (16, including 12 competitors). This distinction corresponds to that made between “weight trainers” and “bodybuilders” by earlier authors such as Bednarek (1985) and Monaghan (2001). We propose to account for the differentiation of these two groups by focusing on the temporality of the processes in which one can identify important moments that enable the conversion to bodybuilding and the shift from outsider to insider status. It is important to understand this shift because it is central to explaining the changes in norms and the moral disengagement that favor the use of chemical substances.

#### Indifference as a Reminder of the Local Order

Our experience as ethnographers is akin to that of “newbies” discovering the singular universe of the gym and its symbolic order. The hall appears as a clearly partitioned space. The equipment is organized into the spaces devoted to cardio-training and muscle-building, which are divided into two zones, one for machines and one for free weights situated in front of the mirrors. In addition to their remarkable physiques, the bodybuilders identified are distinguished by their quasi-exclusive use of the space at the end of the gym in front of the mirrors, which contains the exercise benches, barbells, Olympic bars, disks, pullies, and some machines. This spatial geography has important effects on the rules governing interactions between groups of practitioners with different profiles (Klein, 1993; Andrews et al., 2005) and can create unease for novices who consider themselves slighted (Sassatelli, 2010). It is also understandable why our attempts to make contact for interviews were unsuccessful. There is a real divide between “ordinary” practitioners and bodybuilders that is also manifest in the discourses of the gym-goers. Fabrice (which is not his real name – to protect our interviewees’ anonymity, all first names have been changed), for example, said: “They are two completely different worlds. You can’t compare them; it’s like a two-horse race and Formula One: two different worlds.” The moment of discovery of the training gym corresponds to the identification of a *local order* (Goffman, 1983) that reminds every outsider of his subordinate status.

#### The Shared Enchanted Experience of the Body

None of the practitioners had imagined becoming bodybuilders or using APEDs. As one of them said: “I never thought I would take any, not even proteins.” The initial motivations of Guillaume seem fairly conventional: “I started out with the aim of playing some sports, losing a bit of weight, getting myself into shape. But no way was [bodybuilding] competition a requisite.” And the same is true for Aude, who holds a significant award in the discipline: “Well, lifting weights was initially not at all what I wanted to do in life. And then, suddenly, through training, you find you have a taste for it and you get more and more hooked.” What happened to Aude and other bodybuilders at the gym that explains the shift in their motivation? They wanted to get fit and to lose a bit of weight, and ultimately gained a passion for muscle that drove them to use APEDs. Their disenchanted, rational and utilitarian involvement turned into an enchanted practice. Observed by Weber (2002), disenchantment in modern societies weakened traditional social communities. However, there is also much evidence of re-enchantment in various fields (Suddaby et al., 2017). While most of the gym-goers remain, as in other domains of culture, just “simple consumer-spectators” (Lahire, 2014, p. 75), some of them shift progressively to the “closed order of bodybuilding” (Brown, 1999: 84). As with religion (Suaud, 1978), enchanted body experiences have a fundamental role in explaining future conversion to bodybuilding and are associated with moments of grace by some of our interviewees: “I went into a gym and felt my life had changed; I felt that something was going to happen […]. I said to myself, ‘Ah, that’s it, now I feel something new is happening in my life’ […]. It was this first step into the gym that shook me up” (Igor). Beyond bodily sensations or the embodied pleasures of vibrant physicality, some practitioners describe a kind of “revelation” about the practice of bodybuilding that is characteristic of a moment of conversion and needs to be better understood. Kevin describes a similar feeling and declares: “I was eighteen, I had no other activity, it was my…. I had a life that was, yeah, that was centered on my work and the other jobs I did alongside it […]. My life was empty. I had no hobbies. And training provided something with which I could identify […]. Geez, I’ve never felt as good as that in my life.”

The analysis of the socio-cultural profiles of our sample population brings to light two different social determinants that explain the intention of the practitioners, after a phase of discovery, to continue on the path of bodybuilding. These two groups that emerged stand out, and the individuals interviewed are distributed between them. Some of them may lie on the border between these two categories and cumulate factors conducive to conversion. The first group is characterized by practitioners having an “initial social disposition” to appreciate muscle and strength *via* family support, occupational aspirations or a favorable work environment. Coming from the working classes, they are often active in security occupations and perceive muscular bodies as resources for their work. For example, Quentin states: “It [bodybuilding] makes me look good in my clothes, my security clothing that I wear every day […] I feel that I fill the uniform.” Another example comes from Guillaume, who works in a jail and states: “It interests them, because after all, in prison […], they [the inmates] are all in the fitness room, pulling on weights and so on. So, for them, it’s a point of recognition; they can admire it.” For individuals wishing to use their body as capital, gaining muscle fits well with the recognition inside and outside the gym. Their occupational and familial environments valorize strength and muscle. These practitioner profiles have a lifestyle associated with a working-class habitus as “a set of basic, deeply internalized master-patterns (dispositions) which may govern and regulate mental processes without being consciously apprehended and controlled” (Bourdieu, 1984a). It means that they have internalized “schemes of perception, thought and action” through their socialization (Bourdieu, 1989) that guide the use of their bodies as social resources. As a consequence, the consistency between their practice and their lifestyle reflects a kind of “consonance” (Lahire, 2008).

The second group, described as introspective, is socially more diverse and corresponds to individuals whose life courses are marked by breaks and who have no particular social disposition to valorize muscularity and physical power. Moreover, for them, it is a somewhat separate experience unconnected to their social universes. They are in a kind of “dissonance” corresponding to a cleft habitus that “divides up the different cultural practices and preferences of individuals across all classes” (Lahire, 2008, p. 166). Practitioners with these profiles feel fragilized in their personal and/or occupational lives and soon come to see bodybuilding as a way of attending to their bodies, strengthening them, taking a new grip on life and themselves, and *reinventing* themselves (Kaufmann, 2004). For example, Kevin states: “My life was empty. I had no hobbies. And training provided something with which I could identify […]. Geez, I’ve never felt as good as that in my life.” Similarly, Beatrice states: “I’ve never had an easy time, never in all my life, and I would say it’s the best medicine for me.” For those using it as a resource to cope with a difficult life course, after a divorce, unemployment or other negative social experiences in their life course, changing their bodies, controlling them, being recognized for their personal qualities is an extremely positive experience. Few of them were using their bodies as resources for work. They rather “treat the body as an end in itself,” as the privileged classes frequently do (Bourdieu, 1978), but under the influence of the enchantment of bodybuilding, they may also perceive the body as a resource. However, similar to the “dysmorphic disorders” mentioned above, these social dispositions do not themselves suffice to explain why ordinary people turn to APED use to gain muscle volume, but they combine with the interactions at the gym to explain the conversion to bodybuilding. Even if the paths to bodybuilding can be dissociated upstream, the mechanisms of adherence in the gym are similar.

#### Interactions in the Gym or the Sociological Processes of Election

##### Proving one’s investment: sacrifice for bodybuilding

The wish to use one’s body as capital or as a resource to cope with a difficult life course has a large influence on the way one works out in the gym. The practitioners who are pursuing a bodybuilding career sustain a particular relationship with pain. They have learned to love to hurt themselves. The visibility of the pain is central because it has a phatic function that creates ties, and in Goffman’s word (1967, pp. 90–91), “the gestures which we sometimes call empty are perhaps in fact the fullest thing of all.” Sharing a similar training, pain, and more broadly, lifestyles increase attention and favor mutual recognition. Hervé, who is in the conversion phase, valorizes his “hardcore” training as “an absolute discipline.” He valorizes not only his ability to hurt himself – “when you’ve given yourself a deep work-out, it hurts so much you can’t do any more” – but also his ability to enjoy the pain: “If you don’t love pain, you won’t even get any pleasure […]. The two things always go together. You have to understand that pain is pleasure.” The converts are characterized by this particular relation to pain that “is part of the game, […] you take it as a friend” (Igor). Guillaume and Béatrice describe this paradoxical sensation: “You hurt yourself, but it feels good”; “The muscular pain you get from those efforts is actually a pleasure for me, and I need that pain.” The sensation of the body being pushed to its limits is made concrete in pain. That is what the converts are looking for. The violence of the effort gives them the feeling of existing through pain (Le Breton, 2006) and rediscovering a meaning to their lives by shaping themselves through bodybuilding. All the converts also had identified themselves by the regularity of their gym-going. They all mentioned a regular, committed investment in the practice to explain their admission to the community of bodybuilders. Béatrice states: “I always worked very, very hard […] I always trained a lot,” and Igor’s assiduity is impeccable: “It’s quite simple, from the age of eighteen I don’t think I’ve missed a single week.” On average, the converts train regularly between four and seven times a week, as compared to one to four times for the ordinary practitioners interviewed.

Sacrifice for bodybuilding is one of the necessary conditions. To win attention and thus be able to enter the reserved spaces, one has to accept the growing hold of bodybuilding on one’s life. Beyond regularity in training and gain in muscle volume, one has to show one’s merit through intense investment and corresponding physical effort. This is what breaks the feigned indifference of the experts. They seem to ignore the newbies but, in fact, observe them in what Goffman (1963) calls an unfocused interaction. Through their tacit monitoring they identify the ones who seem worthy of interest. If the latter are sufficiently committed, they begin to be recognized by the restricted circle of “real” bodybuilders, and more than induce these signs of recognition from insiders, our results show that pre-converts are receptive to it. As Kevin testifies, “I got to know a guy like that, in his early thirties, an intellectual, but really well-built, and then he took charge of me. From then on, for 6 months, I came every day of the week, every single day.”

##### Social and mutual recognition

When the experts give their first signs of interest in a practitioner, they validate the first stage of social recognition. The gym managers and the coaches then follow them up in confirming the election of talent. For example, Léo believes that the encounter with his trainer was “the key moment in my career and my success.” Thanks to their legitimate authority, the most experienced bodybuilders valorize some of the would-be bodybuilders by devoting close attention to them. Dolorès was talent-spotted by her trainer: “At the time, the boss of the gym was a world-famous bodybuilder who began to see that I had a taste for it and was training well, that I had the right postures and showed commitment.” Through her investment, Dolorès had prepared for her election. She won recognition in the gym, and above all, she started to assign value to the perspective and the words of the experts in the gym, who became people who mattered and who would later play a decisive role in the “moral disengagement” that eased the transition to APED use.

To enter the bodybuilder community, there has to be cooption and mutual recognition. These perceived signs have a non-negligible influence on interactions in the gym because the community of converts, following the rule of the Three D’s – “dedication, determination, discipline” (Fussell, 2015) – valorizes committed practitioners and/or ignores the dilettantes. Igor, a hardened bodybuilder, easily distinguishes the heterodox individuals not intrinsically motivated by bodybuilding: “I can spot straight away a guy who wants to make progress just for the beach, or a guy who wants to make progress because he’s really got it in his blood. I can tell them apart.” Hervé underscores this point of view and clarifies the distinction: “I don’t much like the people there [in the gym] because there are guys who come just to wander about a bit and play around with the weights, but they are not really concentrating.” The converts conceive bodybuilding as a “serious leisure” (Stebbins, 1992). The competitor bodybuilders are infused with a doxa that enables them to assert and display a strong and shared internal athletic identity. Their work of reimposing symbolic order is that of purists for whom, like artists (Bourdieu et al., 1991), bodybuilding is an end in itself, which explains their contempt for “beach boys.” And the mechanisms of initiation and conversion to the practice of bodybuilding are based on this encounter between persons disposed to this and the guardians of orthodoxy. Their interactions consecrate this conversion. The best specialists show the value they set on the work accomplished. This attention is perceived as a form of strong recognition playing the role of a positive interpersonal ritual. Alfred testifies: “When you have reached a certain level, you start to see the recognition in how people look at you. Ah, it’s nice. Listen, all human beings, we all like recognition, a clap on the back, I mean, you just feel good.” For individuals undergoing conversion, in search of occupational recognition or fragilized by situations of social isolation, this unhoped-for recognition consecrates these individuals by making them members of the subculture (Klein, 1993) and their “new” personality “one apportionment of the collective mana” (Goffman, 1967), defined as what makes the value of things and people (Mauss, 1985). They are honored by the recognition of their qualities and by the treatment as *sacred objects* (Goffman, 1967). Thus, bodybuilders transform the frame of the gym by “keying” it (Goffman, 1974, pp. 43–44), which means that they change the meaning and the experience of the situation. Igor describes the gym as “a second home,” Kevin calls it a “place of meeting and very intense friendship,” and Guillaume changed his self-perception: “*People looked at me like a God, yes, it’s really nice, it’s great…. Yes, I’m the biggest of the gym.*” So, in this context and regarding the social vulnerabilities (Castel, 1991), it is not surprising that the hold of bodybuilding grows on these individuals and gives a strong feeling of social solidarity. The interactions at the gym create an enchantment because the two different social properties, professional situations valorizing muscles and social precariousness, prepare one to be sensitive to the recognition due to the interactions with peers in the gym. It produces a real enchantment. You are no longer just a gym-goer; you become a “real” bodybuilder. As a consequence, resistance to APEDs is weakened for the two types of social profiles we observed and the normalization of APEDs confirms simultaneously the strength of the enchantment that provoked conversion. Whereas the use of supplements is common among all the interviewees in our sample, the use of APEDs represents a frontier between “ordinary weight trainers” and “bodybuilders.”

### Effects of Conversion to APED Use

#### Practitioners’ Relationship With Doping Substances

Only converted practitioners who commit themselves assiduously and are integrated into the bodybuilding community will be led at one point or another in their trajectory to consider the possibility of using APEDs. The slippage of representations regarding doping substances and the actual use of them then become possible. As with the triggers of conversion to bodybuilding, it can be observed that the tipping-point into doping is linked to interactions in the gym with trainers or practitioners. Some bodybuilders follow the advice of their trainers. For example, Guillaume states: “He [his trainer] saw me and then he was like: ‘But have you ever taken substances?’ And I’m like: ‘No, never,’ and he’s like: ‘OK then, we’ll start gently.’ And, it’s true, we started with very small doses and then they went up, up, up.”

On the margins of the ordinary world, the bodybuilding community constitutes a real unifying *subculture* with its own norms (Klein, 1993; Lowe, 1998; Monaghan, 2001; Bunsell, 2013). Pierrick’s impression of his conversion to bodybuilding illustrates this shift clearly: “It’s a bit like entering a new world.” Conversion is accompanied by an evolution of the relation to APEDs. In this subculture, substance use is commonplace and even seems unavoidable: “Anabolic steroids and stuff like that. Yeah, yeah, of course. You get into that, you have to, unless you want to finish eleventh out of ten, you have to, because everybody does.” Igor states: “If I want to keep up with what is asked of me, I have to use them.” Aude explains that unlike other sports cultures where doping leads to strong symbolic discrediting, there is little or no ethical condemnation in the bodybuilding world because APEDs are part of the culture. Samuel declares: “As for cheating, well, put that in big quotes, because if there are ten of you, and all ten… all ten take the same substances, sorry but I don’t see how that is cheating.” The diffusion of doping in the milieu does not affect the equity among rivals in competitions.

Insiders relativize the effect doping can have on their performance. Their representations of doping contrast with common understandings. Igor strongly rejects the widespread, simplistic idea that substance-taking means a direct gain in muscle volume: “People think you just take a shot and then you swell up all over and there’s nothing more to do. That’s a really dumb idea. You can take thousands of products and if you don’t train, nothing happens.” There is a kind of “team representation” (Goffman, 1973) among the insiders interviewed that enables them to legitimize their choices for the interviewer. They present access to doping substances as something that has to be earned and combined with real expertise: “I’ve learned about nutrition, training, and how to train very clearly. It means pushing yourself to the limit. When you’ve got at least that basis, then you can try out these products” (Alex). For them, doping in no way devalues performances or the merit of the work done. Discourse analysis shows that these bodybuilders neutralize the conventional moral norms that associate doping with deviance. Their progressive entry into the bodybuilding world is accompanied by a banalization of doping substances. However, APED use divides views of the “authenticity” of muscle (Sassatelli, 2010). For “ordinary” gym-goers, authenticity means the absence of artificial, external aids, whereas the converts to bodybuilding believe that the use of doping products does not disqualify their performance and their muscle volume therefore remains authentic. This recurrent reaffirmation of the authenticity of performances despite the aid of pharmacology restates and strengthens the doxa held by the bodybuilders regarding doping. Therefore, there is a kind of paradox: from an external point of view, “body built” muscle presents the image of artificial muscle because it is associated with doping, but from an internal point of view, it is judged authentic by the bodybuilders. In the interviews, the bodybuilders’ insistence on minimizing the effects of doping substances on their level of performance confirms their determination to make their muscle authentic. Conversion to bodybuilding thus induces a legitimation of APED use, and this legitimacy also changes perception of the risks.

#### A Sense of Controlling the Risks of Doping

The bodybuilders interviewed have developed a sense of controlling the risks with their insider know-how, their medical knowledge and practices, and their own bodily sensations.

##### Insider knowledge

The bodybuilders clearly understand the physical risks they are taking by doping. Alex describes the case of his brother: “My brother’s coach messed him up. Now he’s got gynecomastia, and what with that and his loose skin all over… as for his libido, I think there’s nothing left, so altogether…. As for myself, I pray to heaven it won’t happen to me.” Alfred says he has known lethal cases: “I’ve known people who have taken it, and now they’re dead. At the gym I went to, there was a young man who took ephedrine and had a weak heart and now he’s dead […] His heart couldn’t take it. It just gave up.” However, they have developed insider expertise on the fringes of evidence-based medicine, which gives them a sense of controlling the risks. Some of them, like Igor, seek out information: “I found out a lot, I read a lot about products, which ones work, the side-effects, which one would be best for what I wanted to achieve, while limiting the damage, you know […], a lot of information from the Internet or books.” Others turn to people with expertise. Aude takes “advice from people who are already in the scene,” and Pierrick assembles a lot of information from the community: “I didn’t do it all alone, either […]. It was all done on information from people who know the area well. You talk to them, you inform yourself.” As observed in cycling (Brissonneau et al., 2008), interactions with more experienced peers make the initiation into APEDs possible. After being recognized by the gym’s local experts, gym-goers become “real” bodybuilders and have access to the local expertise in training, nutrition, etc. They trust the trainers or coaches who they consult for their expertise on the different products. How they share their practices and knowledge not only helps them to prepare the competition but also reactivates social solidarity and mutual awareness creates strong ties. Thus, for Guillaume: “It was always the coach who had the knowledge and told me what to take, how, on which days, at what time.” Dolorès also entirely trusts her coach: “It was my coach […], a healthy person who gives good advice […] I’m not going to mess around taking this or that, that way or this, because I don’t know how, and I’m not going to go looking for information on the Internet. I’m too afraid of doing something stupid […].” Guidance is also given about the right way to administer the substance. Injection is not an everyday activity; it has to be learned. Igor relies on a friend who recommends products: “It was […] a friend with more experience who gave me my first injection, because I didn’t know how, and I didn’t want to screw up, it’s important.” Likewise, Guillaume informs himself about what precautions to take: “So, he told me afterward that the green syringe was for taking up the liquid and then you take a new one to avoid wounds and then infections […]. I was told how to measure so as to avoid the sciatic nerve […], not to limp like a cripple for a week […]. And then I tried it, and it came naturally.” The experiences and interactions with more experienced users gave a sense of developing expertise regarding APEDs, a “knowledge” that is transmitted among bodybuilders, from generation to generation, and gives the athlete the sense of controlling the risks.

##### The uses of medicine

In line with Monaghan’s (2001) observations, this sense of controlling the risks is sustained by medical knowledge and monitoring. All the interviewees who stated that they use APEDs said that they keep watch on their health as well as they can by referring to medical knowledge. They compare and feed their insider knowledge with medical knowledge, so they turn to this expertise to oversee their state of health and regulate their substance use. For them, the worlds of medicine and bodybuilding are not totally impermeable since there seem to be connections, as Ubert testifies: “A well-known gym manager said to me: ‘We’ll go and see the doctor.’ He was an old, very kind doctor who makes an analysis, checks that you have no problem, and, I can remember, it wasn’t even testosterone, it was Deca-Durabolin, a very, very soft drug, and then Primobolan.” For some bodybuilders, this medical monitoring even seems to be a prerequisite for their use: “If you want to go in for high-level competition, you have to use some products. But it has to stay medicalized and 100% controlled” (Pierrick). Guillaume is monitored by a sports doctor: “I have a sports doctor and I tell him everything. We always do a check-up before and after a competition, a full check-up with blood sample, heart ultrasound and ECG. That was a point of honor, and I said, I’ve always said, ‘If someone tells me there’s any kind of problem, I’ll stop everything”’. This link with doctors supports the sense of controlling the risks, even if there always remains some uncertainty for Alex: “All the same, we are still playing with our lives […]. The aim is do the best we can, I’m monitored by an endocrinologist, I often do a PCT […]. In the end, it’s clear, you can’t control everything 100%, but at least you can take basic precautions.” The prescribing doctors are a reassurance. They make it possible to avoid purchasing through the Internet or on the black market, which can lead to anxiety about the quality of the products. This is true for Béatrice regarding the traceability of APEDs: “I know that the products he supplies come from Swiss laboratories, so for a start, there are no customs problems, and I know there is quality control. It’s pharmaceutical quality. I don’t want anything else.”

##### The register of physical sensations

Bodily sensations are the third element on which bodybuilders rely to control the risks. The effect of the products can be felt: “Testosterone works, it really does. Chiefly in terms of training, you have better congestion, a better physical feeling, that’s clear, there’s much less fatigue, speedier recovery between two sessions or at the end of the week” (Dolorès). These physical sensations are described as out of the ordinary by some interviewees: “The feelings are rather extraordinary, I mean, wow, you feel great” (Léo); “Yes, it’s extraordinary. No, really […] there are no other words for it, I mean, you have great recovery, and the amazing thing is, you never get ill. […] You have speedy recovery, impressive strength” (Alex). APEDs thus give the sense of being able to reduce the risk of injury: “These products also give better recovery, and they prevent all the micro-tears you can get from extreme effort, because after all they are extreme efforts” (Dolorès). These new physical sensations are associated with benefits and give a sense of well-being and power. The feeling of being in good health is based on the benefits of these new sensations. Alfred describes them as follows: “You feel… you know… you’re in good health all the same, I mean.” The bodybuilders gain confidence as their experience grows in parallel with their new physical power due to doping and the sense of being in control of the risks. Sometimes the negative secondary effects soon make themselves felt, as they did for Igor, who describes his reaction after using clenbuterol: “But that was something that didn’t suit me, it gave me a headache and my heart was racing. I didn’t much like that. So, I try to respect my body while going for performance.” These alarm signals did not, however, call into question his whole pharmacological program; rather, they led only to a readjustment. Like alchemists, bodybuilders try out substances, observe their effects and adjust the dosages according to their experiences. Like the marijuana users studied by Becker (1963), our interviewees show themselves to be particularly attentive to their sensorial experiences, and their abilities to perceive the effects of substances confirms for them their sense of being in control of the risks. The body as a tool of “knowledge” makes it possible, through the personalization of APED use, to have the sense of adapting the substances to one’s own physiology: “You can handle it, you can totally handle it, that is chemistry too, physiologically, you have to be very, very careful, some things are right for some people, not for others. And then, you adjust and then – whatever people may think –without having to take enormous quantities. What I mean is, that’s not what makes the difference” (Aude).

The knowledge and advice acquired within the bodybuilding community, the monitoring by the medical corps (doctor, endocrinologist), and the degree of attention paid to bodily sensations, reinforce bodybuilders’ sense of controlling the risks to their health. These three justifications, found in the interviews, enable us to give an account of their “rational” approach to understand and then explain some behaviors that are judged as transgressive and therefore morally reprehensible according to the dominant societal values.

## Discussion

By concentrating on an analysis of bodybuilders’ social configurations and lived social experiences and having the bodybuilders speak for themselves, one is able to contextualize their relation to APEDs. This qualitative research enriches the explanatory models based on causal explanations (Petróczi, 2007; Barkoukis et al., 2011) and on the way in which moral disengagement reduces resistance to substance use (Boardley and Grix, 2014). It makes it possible to observe the rooting of moral disengagement in the practitioners’ social environment. It can be seen that the different mechanisms of moral disengagement identified in the literature are not clearly expressed through our collection of data. For example, it is difficult to speak of a “displacement of responsibility.” For the interviewees who declare that they consume doping substances, this is not really a culpable activity for which someone else has to be incriminated. There is indeed a “diffusion of responsibility,” but this argument is not used spontaneously to justify substance use. In our study, APED use is undertaken because it is a shared norm within the restricted circle of “real” bodybuilders. The fact that bodybuilding is not regarded as a “real” sport and is stigmatized because of doping leads the interviewees to make “advantageous comparisons” when they describe their healthy lifestyle (regular physical activity, controlled nutrition, no alcohol, sleep management, etc.), as for example: “Is it better to drink three liters of wine a day and eat fast food than do bodybuilding?” (Aude) and “We were all a bit afraid to begin with, but I say myself, it’s no more dangerous than getting drunk every night, or smoking, or guzzling hamburgers every day. I think it’s no more dangerous than that […]” (Léo). Comparison with other elite sports enables bodybuilders to point out that bodybuilding is a sport like any other, demanding major efforts, talent and merit. As Léo explains: “We know it’s high-level competitive sport, and they all do it. I mean, let’s not kid ourselves, do you think that people like Rafael Nadal who play tennis for three hours in forty degrees are running on water?” (Léo). Guillaume states: “Because… cycling, that got busted, but they still film the Tour de France, and you know that even the winner must be taking stuff” (Guillaume). Finally, the “distortion of consequences” is observed in two complementary, linked modalities: modification of conceptions of health and positive body experience. In other words, it is the immediate time, felt in positive body experiences both in terms of physical sensations and at the level of recognition, that is most important and not the longer-term perspective of the anticipation of risks or the time of epidemiologists and health experts. Thus, our results echo those of research based on the life-cycle model, which underscore the importance of taking temporality into account in order to understand doping (Petróczi and Aidman, 2008). Moreover, our findings question the opposition between the figure of an individual “unaware” of the risks and the actor who rationalizes his pharmacology in a cost/benefit calculation and shows that these are the experiences that transform the relation to APEDs through experience and learning. Our interviewees do not anticipate their careers; they do not start out with performance objectives and do not progress in a linear way through the various phases and cycles described by Petróczi and Aidman (2008) (choice, goal commitment, execution, feedback, evaluation, adjustment). It is important to state that our observation is over a longer time-scale than that of life-cycle model studies, and while situational and environmental factors are present in the life-cycle model, our singularity has been to observe how the combination of the “ecology” of interactions in the gym, analyzed through an ethnological approach, and the initial social conditions evolve over time and determine how individuals adopt new bodily practices. These observations reinforce the argument that bodybuilders’ doping practices have to be understood as “activities performed along a continuum of cultural and societal (over)conformity, rather than actions representing societal abnormality” (Andresasson, 2013). According to this statement and our findings, it seems that the tools used by sociopsychologists to observe morality are relevant; however, labeling it as a disengagement is problematic. Thus, as researchers, we may describe these bodybuilders as if their morality is weakened. However, the analysis of their actions in context shows that they just deviate from a social norm in a “positive deviance” (Hughes and Coakley, 2013) that conforms to the bodybuilding subculture. Although they put their health at risk, they are simply adapting to the bodybuilding subculture and do not damage any other competitors. As a consequence, the normalization of APEDs in bodybuilding is not really a moral disengagement; rather, it is a moral shift. However, this shift due to conversion is not a fixed state. Beliefs and practices vary over time, with moments of instability, moments when bodybuilders have the sense of controlling the risks – situated rationalities – and moments more marked by reflexivity and doubts. In contrast to other conversions that may be more durable, such as spiritual conversions, in the bodybuilding conversion, bodybuilders may be confronted with a disenchanted experience of the body. While at some moments, bodily experiences may reinforce a sense of control of one’s health, at other times, the perception of a fragilized body can create a tension between beliefs and the tangible effects of the substances. This reflexivity is greater when there is a mismatch between practices and the perceptions that the person has of them, when the external gazes that count are combined with discrepant bodily sensations. In these circumstances, the disenchantment of the milieu and questioning of one’s position within orthodox practices may take place through doubts about the effects of APEDs or a loss of confidence in APED prescribers.

This study also has some limitations. The first is that we did not address the question of gender, although our sample is mixed. This was a deliberate choice so as center the focus, but even if women are more often “dependent” than men, their conversion processes are analogous and their adoption of “manly” codes is not different even if it is even more difficult to undertake. A second limitation is that we did not use a method to measure levels and types of moral disengagement. This was indeed not the initial ambition of this study, which aimed to be resolutely complementary and articulated in relation to the existing literature. Indeed, our goal was to bring to light the social mechanisms underlying the normalization of APEDs. Therefore, we suggest that subsequent studies utilize a disengagement moral measure to reinforce our findings. However, we also identified some obstacles that contravene the implementation of a mix-methods study. On one hand, bodybuilding is not widespread in French-speaking parts of Switzerland, and a consistent sample was quite impossible to achieve. On the other hand, gym managers did not welcome us for the inquiry. Thus, asking their clients to complete a questionnaire had little chance to be accepted, and answers would certainly also be under the influence of a social desirability bias. It may have also created mistrust that would not fit well with ethnographic observations and interviews. Finally, we wished to explore the diversity of the respondent profiles so that the interviews focused on career that covers a range of 3–40 years of experience in the gym. Thus, another sample would be needed to use questionnaires to observe how morality changes over time. However, in the future, with better research conditions, we suggest using mixed methods.

## Conclusion

Appearance- and performance-enhancing drug use is an object of study that requires one to articulate several scientific approaches. In this study, we mobilized a sociological approach centered on the process of conversion to bring to light the role of the social and cultural variables that give a better understanding of how a person becomes a bodybuilder and can be led to use APEDs. Our sociological approach and our results complement the explanatory models offered by psychology and invite discussion of the scope – and limits – of causalities constructed on the basis of correlations. For example, a critical stance toward explanations in terms of pathology is not equivalent to stating that no pathology exists. However, given that the questionnaires are answered by gym-goers, most of whom are already bodybuilders, it is possible that the pathology may be produced by bodybuilding and is not necessarily its cause.

We have brought to light some social determinants that may explain why people undertake intensive bodybuilding, we have shown the impact that interactions in a gym can have on the practitioners’ development, and we have considered how risk and bodily experiences are related to the effects of APEDs. However, no single level of analysis can explain why some individuals become APED users. It is the combination of these ingredients that is the determinant. Approaches that are both macro and micro and both psychological and sociological are complementary. Each axis of analysis helps to explain some of the factors and one aspect of this complex question. We could consider the topic even further and suggest for future research that instead of focusing on pathological aspects of bodybuilding, research in psychology could make a significant contribution on habitus. Habitus is analyzed as social memory, especially because of its focus on the role of families’ socializations. However, it should be extended to a much broader memory that includes emotional and cognitive experiences in their more psychological understandings. The dispositions generated by the habitus are shaped by experiences that are not divided into the disciplines of psychology and sociology because they are simultaneously both. As Reay (2015) recalls it, the concept of habitus “allows us to better understand how the psyche is formed in and through the social.” Further research would certainly benefit from a research design that could combine sociology and psychology. Bourdieu (2000) already proposed to unite the strength of sociology and psychoanalysis. However, we instead suggest achieving his proposal with other psychological approaches that could analyze how social and psychological variables of careers combine. Nevertheless, although the pertinence of pluridisciplinary approaches is often asserted, studies that take these approaches are few. The logic of the production of research is disciplinary but the real world is not – instead it is at once psychological, social, economic, legal, etc. Moreover, by focusing on situations, lived experiences and their variations, and even considering contradictions depending on the moment in the bodybuilders’ careers, this work makes it possible to better prepare strategies for communication and support in the framework of doping prevention.

Finally, in this article, we have aimed to emphasize the importance of the temporality of processes. This enables us to better articulate the models (cf. **Figure 1**, which is a synthesis of our data and the literature). If social and psychological determinisms influence one’s arrival in a gym, they combine and interact over the course of time. Instead of understanding morality only at a given time, observation in the gym and analyses of the accounts of life courses show us how it is progressively constructed.

## Author Contributions

RC did his Ph.D. on the topic. He was supported by a FNS grant that was obtained by FO. RC collected most of the data with a minor contribution of FO. FO and PR supervised RC for his Ph.D. FO and PR wrote the manuscript in collaboration with RC.

## Conflict of Interest Statement

The authors declare that the research was conducted in the absence of any commercial or financial relationships that could be construed as a potential conflict of interest.
